# Supplementary material for: Seminoma and Embryonal Carcinoma Footprints Identified by Analysis of Integrated Genome-Wide Epigenetic and Expression Profiles of Germ Cell Cancer Cell Lines
Source: PLoS One. 2014 Jun 2;9(6):e98330. doi: 10.1371/journal.pone.0098330 (PMC4041891; doi:10.1371/journal.pone.0098330)
Supplement: File S1 — ZIP file containing DMRforPairs output for significant regions. Please start from the html files. (ZIP) [file pone.0098330.s008.zip › figures/32841.pdf]

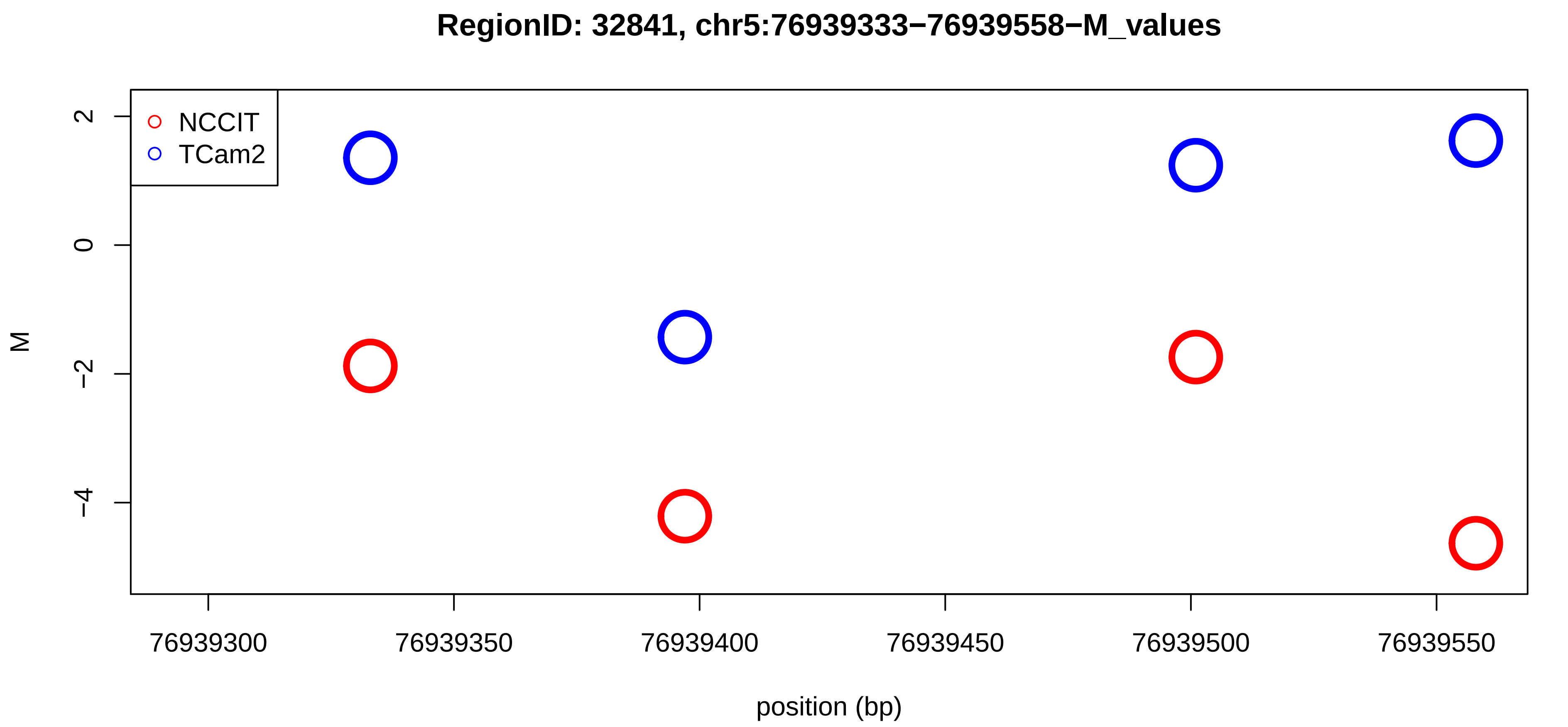
RegionID: 32841, chr5:76939333−76939558−M_values
M
2
0
−2
−4
NCCIT
TCam2
76939300
76939350
76939400
76939450
76939500
76939550
position (bp)

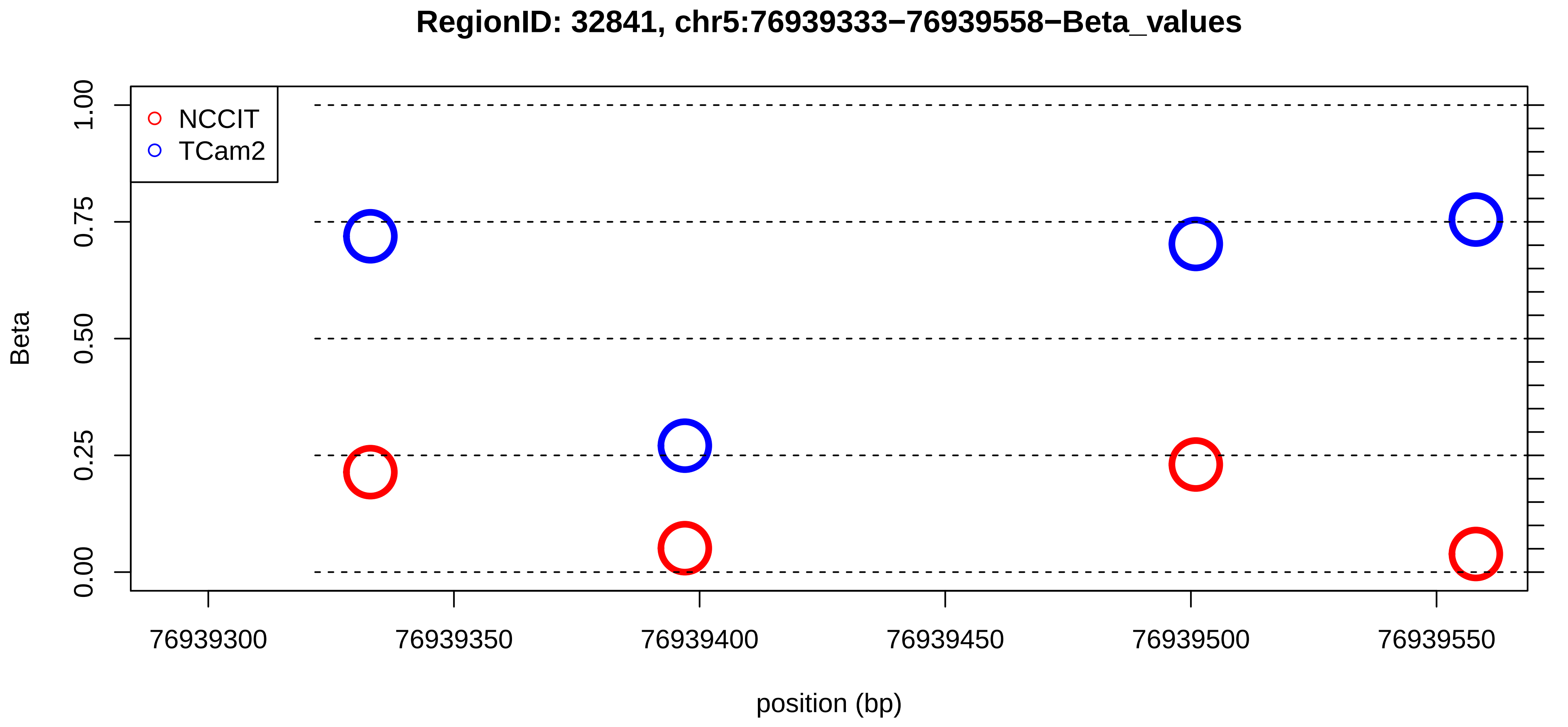
RegionID: 32841, chr5:76939333−76939558−Beta_values
Beta
1.00
0.75
0.50
0.25
0.00
NCCIT
TCam2
76939300
76939350
76939400
76939450
76939500
76939550
position (bp)
